# Neurophysiological and neurocognitive mechanisms underlying the effects of yoga-based practices: towards a comprehensive theoretical framework

**DOI:** 10.3389/fnhum.2015.00235

**Published:** 2015-05-08

**Authors:** Laura Schmalzl, Chivon Powers, Eva Henje Blom

**Affiliations:** ^1^Department of Family Medicine and Public Health, School of Medicine, University of California San DiegoLa Jolla, CA, USA; ^2^VA San Diego Healthcare SystemLa Jolla, CA, USA; ^3^Center for Mind and Brain, University of California DavisDavis, CA, USA; ^4^Department of Clinical Neuroscience, Karolinska InstitutetStockholm, Sweden; ^5^Department of Psychiatry, University of California San FranciscoSan Francisco, CA, USA

**Keywords:** yoga, movement, breath, attention, allostatic load, basal ganglia, bottom-up, top-down

## Abstract

During recent decades numerous yoga-based practices (YBP) have emerged in the West, with their aims ranging from fitness gains to therapeutic benefits and spiritual development. Yoga is also beginning to spark growing interest within the scientific community, and yoga-based interventions have been associated with measureable changes in physiological parameters, perceived emotional states, and cognitive functioning. YBP typically involve a combination of postures or movement sequences, conscious regulation of the breath, and various techniques to improve attentional focus. However, so far little if any research has attempted to deconstruct the role of these different component parts in order to better understand their respective contribution to the effects of YBP. A clear operational definition of yoga-based therapeutic interventions for scientific purposes, as well as a comprehensive theoretical framework from which testable hypotheses can be formulated, is therefore needed. Here we propose such a framework, and outline the bottom-up neurophysiological and top-down neurocognitive mechanisms hypothesized to be at play in YBP.

## Definition of Yoga-based Practices in the Context of this Paper

During recent decades numerous yoga-based practices (YBP) have emerged in the West. According to recent surveys, yoga is practiced by over twenty million people in the USA alone, with its status having evolved from a niche activity to the catalyst of a blooming multimillion dollar industry. Yoga is also beginning to spark growing interest within the scientific community, and a rapidly increasing number of studies are investigating the effects of yoga on physiological parameters, perceived emotional states, and cognitive functioning (Gard et al., 2014a). At the same time, the recent “yoga boom” has been met with controversy, especially when it comes to defining yoga in modern contexts. For example, in the West yoga has become virtually synonymous with posture or movement-based practice (Feuerstein, 2003). However, this type of practice is a relatively recent phenomenon, and according to some sources there is little evidence that movement-based practice has ever been the primary aspect of any ancient Indian yoga tradition (Singleton, 2010).

In its traditional sense, yoga is considered a spiritual practice with roots in the Yoga Sutras of Patanjali of which there are numerous interpretations and translations (Satchidananda, 1978; Hariharananda Aranya, 1983; White, 2014). In the context of these texts, the various components of yoga such as “asana”, “pranayama”, and the “samyamas” refer to postural, breath-based and meditative practices aimed at directing and refining “prana” (subtle energy or life force), with the ultimate goal of reaching a state of “samadhi” (an evolved state of the human spirit often referred to as pure consciousness). Since our aim is, however, to provide a secular and operationalized definition of YBP that is useful within our current and more limited Western scientific paradigm, we will largely refrain from the use of yogic terminology in this paper. Similarly, we will not focus on yoga from the perspective of a specific lineage, but instead outline a framework for YBP as modern psychophysiological therapeutic practices that employ a series of movement-, breath- and attention-based techniques inspired by a variety of yogic traditions. The main goal of YBP in this context is to optimize health, promote stress reduction and increase self-regulation, from both a prevention and treatment perspective. We suggest that science can contribute by operationalizing the components and carefully evaluating the efficacy of YBP, for the purpose of furthering an evidence-based understanding of their effects and of promoting their integration into mainstream medical practice. In addition, a science-based understanding of YBP can help elucidate the mechanisms that underlie the effects of YBP, so that they can be precisely tailored to target the specific needs of different populations.

In the following sections of this paper we will: Provide a brief and critical review of a subset of the literature on YBP applied in non-clinical populations; Outline the main methodological shortcomings of previous studies and address some important considerations for future research in the field; Provide a detailed description of the main components of modern YBP that we believe need to be taken into account when designing and describing interventions; Outline the main neurophysiological processes and neural circuits we propose to be at play in and influenced by YBP.

## Brief Overview of the Literature Investigating the Effects of Yoga-based Practices

**Figure 1:**
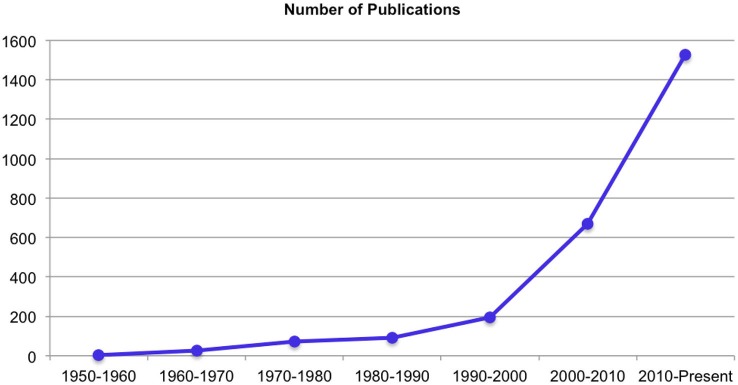
**Increase of yoga related publications over the past decades—PubMed search performed in March 2015 for articles with the term yoga in the title, abstract or keywords**.

Over the past decades there has been an exponential increase in publications on yoga related research (Figure 1). For the purpose of the current paper, we will review a specific subset of studies targeting healthy populations undergoing YBP protocols that involve at least some degree of posture and/or movement. Hence, studies on clinical populations and purely breath-based or meditative practices are not included. We will summarize what these previous studies suggest regarding the effects of YBP on physiological parameters, body awareness, self-reported emotional states and stress, and cognitive functioning.

### Physiological Parameters

Only a small number of studies on healthy populations have investigated the effects of YBP that include postures or movement on physiological parameters such as stress hormones, inflammatory markers and cardiovascular indices.

One of these studies (Rocha et al., 2012) compared salivary cortisol levels in two groups of military populations who underwent 6 months of either yoga or regular physical exercise. Following the program, the yoga group showed significantly lower cortisol levels than the exercise group, but the results must be cautiously interpreted as they are based on a single cortisol sample collected pre and post intervention. In addition, the yoga intervention is only vaguely described as a combination of asana, pranayama, and meditation. Another study (Kamei et al., 2000) involving electroencephalography (EEG) recordings and serum cortisol measurements, found an increase in frontal alpha rhythm and decrease in cortisol after a single yoga session. However, it should be noted that the experimental group was comprised of self-selected yoga teachers who were not compared against a control group. Moreover, the yoga session was not described in sufficient detail to allow for replication of the study.

A study using magnetic resonance spectroscopic imaging (MRSI; Streeter et al., 2007) investigated the effect of a single hour of yoga practice on y-aminobutyric acid (GABA) levels. In an experimental group of experienced yoga practitioners, GABA levels were measured before and after an hour of yoga practice, whereas in a control group of yoga novices, GABA levels were measured before and after an hour of reading. Only the experimental group showed a significant increase in GABA levels, which the authors interpreted to suggest that yoga may be effective for treating disorders associated with low GABA levels such as depression, anxiety and epilepsy. The study was weakened, however, by a lack of random assignment, unmatched interventions, different levels of yoga experience between group participants, as well as an insufficient description of the yoga protocol.

Parshad and colleagues (Parshad et al., 2011) measured a series of cardiovascular indices before and after a 6 week yoga program. The experimental group was a self-selected cohort of medical students enrolled in a yoga course for stress management, and there was no control group. The results indicated reduced total peripheral resistance (TPR), as well as increased arterial compliance (CWK), stroke volume (SV) and cardiac output (CO). Lastly, a pilot study measuring blood pressure (BP) and heart rate variability (HRV) in twelve healthy adults before and after an 8 week yoga program (Papp et al., 2013), found a significant increase in HRV which was interpreted to suggest that yoga promotes increased vagal tone and reduced sympathetic activity. While this interpretation is warranted, the study calls for replications with larger sample sizes and the inclusion of control groups.

### Body Awareness

Body awareness is a multi-dimensional construct that entails a combination of proprioceptive and interoceptive awareness (Mehling et al., 2012). Since most types of YBP emphasize attention to proprioceptive and interoceptive signals, it can be hypothesized that they are an efficient method for fine-tuning body awareness. However, so far only a few studies have directly investigated this potential effect of YBP.

An early study (Rani and Rao, 1994) compared self-reported body awareness as assessed by the Body Awareness Questionnaire (Shields et al., 1989) from individuals who completed a 3 months long yoga program, to entering enrollees of the same program. Though participants who finished the program reported greater body awareness, no pre-post comparisons were done. In addition, the yoga program was insufficiently described as involving a daily asana and pranayama practice.

More recently, David and colleagues (David et al., 2014) assessed body awareness in relation to susceptibility to the rubber hand illusion in advanced yoga practitioners and controls with no yoga experience. On average, yoga practitioners scored significantly higher on the Body Perception Questionnaire (Porges, 1993) than control subjects, but self-reported body awareness did not correlate with performance on the rubber hand illusion experiment. A further study from the same laboratory (Fiori et al., 2014) assessed proprioceptive and vestibular body signals, as well as the presence of self-transcendence (ST) traits in a group of advanced yoga practitioners and controls with no yoga experience. The processing of body signals was assessed via the Rod and Frame Test (RFT; Asch and Witkin, 1948), and ST was measured with a sub-scale of the Temperament Character Inventory (TCI; Cloninger et al., 1994). Overall, yoga practitioners showed higher accuracy in the RFT and higher ST scores on the TCI. The findings were taken to suggest that yoga practitioners have a higher degree of body awareness that may be related to aspects of ST.

Lastly, indirect evidence for the effect of YBP on body awareness comes from a recent study (Villemure et al., 2014) using sensory testing as well as neuroimaging techniques (voxel-based morphometry and diffusion tensor imaging) to investigate the neuroanatomical underpinnings of pain detection and pain tolerance in yoga practitioners and controls with no yoga experience. Although yoga practitioners did not have a higher pain detection threshold, they tolerated pain (as measured by the length of time they kept their hand in cold water) more than twice as long as controls. Yoga practitioners also had more gray matter volume (GMV), expressed as percent of total intracranial volume, in a number of regions including the insula, cingulate cortex, medial prefrontal cortex, inferior and superior parietal lobule, as well as increased intra-insular white matter connectivity. Moreover, insula GMV correlated positively with pain tolerance (left and right insula) and years of yoga practice (left insula only). Self-reports of strategies used to tolerate the pain revealed that yoga practitioners used much more “embodied” approaches (e.g., focusing on the breath, attending to the sensation, observing the pain without reacting etc.) compared to controls (e.g., trying to ignore the pain and distract oneself). Taken together, these findings suggest that increased pain tolerance in experienced yoga practitioners may be a consequence of adaptive insular changes, mediated by increased parasympathetic activity and interoceptive processing.

### Self-Reported Emotional States and Stress

Self-reports of emotional states and stress are a frequently used outcome measure in research on YBP (Riley and Park, 2015).

An early study (Wood, 1993) measured self-reported levels of vitality (e.g., perceived levels of alertness, sleepiness, enthusiasm, sluggishness, calmness, nervousness etc.) in a gentle form of yogic movement and breathing vs. relaxation or visualization techniques. Over a period of 2 weeks, participants took part in two sessions of each modality, and vitality was measured via visual analog scales immediately before and after each session. The yoga practice session yielded higher levels of self-reported vitality, but given the limited number of sessions and the insufficient description of the intervention, the conclusions are not broadly applicable. Self-reported wellbeing as measured by the Subjective Well-Being Inventory (Sell and Nagpal, 1992), was assessed in a self-selected group of health care practitioners who underwent a 4 months long yoga program consisting of five one-hour yoga classes per week (Malathi et al., 2000). Though the participants reported increased levels of well-being by the end of the program, there was no control group to rule out external factors that may have promoted the effect.

A more recent study (Gard et al., 2012), looked at self-reported perceived stress (as well as other psychological outcome measures) in attendees of a 4 months long residential yoga program described as daily sessions of asana, pranayama and meditation, as well as didactic course work focusing on the integration of yoga practices into daily life activities. Compared to a control group of individuals not participating in the program, attendees exhibited lower levels of perceived stress measured with a 10-item version of the Perceived Stress Scale (PSS; Cohen et al., 2009), statistically demonstrated to be mediated by increased levels of mindfulness and self-compassion. The authors appeal to existing models used to explain how mindfulness affects wellbeing (Shahar et al., 2011), but do not offer specific hypotheses of additional/alternative mechanism directly related to their yoga-based intervention.

Lastly, in their study with military populations participating in a 6 months yoga program mentioned above, Rocha and colleagues (Rocha et al., 2012) documented reduced levels of self-reported depression, anxiety and stress.

### Cognitive Functioning

To conclude this brief literature review we will look at studies investigating the impact of YBP on cognition including attention, memory and executive functioning.

One study looking at the effect of a 10 day long residential yoga program on visual attention (Telles et al., 1995), found that overall the experimental group improved in their ability to detect flicker frequencies, whereas a control group who did not participate in the program showed no improvement. These results were taken to suggest that yoga may hone one’s ability to detect subtle changes in visual stimuli, but there is not enough reported detail about the yoga intervention to inform an understanding of which aspect of the yoga practice may have promoted improvement in visual attention. Another study on visual attention (Narayana, 2009) found faster reaction times on a visual color discrimination task in yoga practitioners compared to a group of non practitioners. The findings were attributed to increased alertness and visuo-spatial attention promoted by the yoga practice, yet again the insufficient detail about the yoga protocol prevents an interpretation of the causative factors driving the results. In addition, the experimental and control groups were not matched for size (26 vs. 42 participants), which makes the findings vulnerable to interpretative error.

In regard to memory, Gothe and colleagues (Gothe et al., 2013) found greater improvements in working memory in novice practitioners after a single session of yoga compared to a single session of general aerobic exercise. Improvements in both short-term and long-term memory were also reported in the study on military populations by Rocha and colleagues mentioned previously (Rocha et al., 2012).

As for executive functioning, one study (Manjunath and Telles, 2001) reported improvements in problem solving ability in a group of female school children after 7 days of yoga practice, compared to a group of children performing the same amount of regular physical exercise. Specifically, the children participating in the yoga classes were reported to have come up with more efficient solutions (planning time, execution time and number of moves) on the Tower of London Task (Shallice, 1982). As for most of the previous studies, there are insufficient details about the yoga intervention to draw any conclusions about what might have yielded the problem solving improvements. Another study (Oken et al., 2006) assessed alertness and executive functioning as part of a battery of physical, psychological and cognitive assessments, in over 100 seniors randomly assigned to a yoga, exercise, or wait-list control group. The intervention took place over 6 months, with one led class per week complemented by home practice. Alertness was assessed via quantitative electroencephalogram measurements (EEC), and executive functioning was assessed with the Stroop Task (Stroop, 1935). While no group differences were detected in either outcome measure, the study has to be credited for a well-powered, randomized, and controlled design, as well as a detailed description of the yoga intervention. The authors argue that their findings may be due to ceiling effects, and maintain it is still likely that yoga can positively affect cognition.

More recently, Froeliger and colleagues (Froeliger et al., 2012a) used voxel-based morphometric analyses to compare GMV in experienced yoga practitioners and controls with no yoga experience, and correlated GMV with self-reported errors on a Cognitive Failures Questionnaire (CFQ; Broadbent et al., 1982). On average, yoga practitioners exhibited greater GMV in frontal, limbic, temporal, occipital as well as cerebellar regions. In addition, they reported fewer cognitive failures on the CFQ, indicating fewer errors in attention, memory, and motor function in everyday tasks. Interestingly, increased GMV correlated positively with years of yoga practice, and negatively with self-reported number of cognitive failures on the CFQ. The authors concluded that yoga practice may promote neuroplastic changes in neural systems that support executive functioning.

Another study (Gard et al., 2014b) investigated age-related decline in fluid intelligence—i.e., a set of abilities involved in coping with novel environments and abstract reasoning (Sternberg, 2008)—as well as resting-state functional brain network architecture in experienced yoga and meditation practitioners. Compared to controls who had no experience with either of these practices, yoga and meditation practitioners had less age-related decline in fluid intelligence as measured by the Raven’s Advanced Progressive Matrices (APM; Raven et al., 1998). They also had more resilient functional brain networks, as reflected by more resilience to attacks in the context of artificial neural network simulations (Achard et al., 2006), across cortical, subcortical and cerebellar regions. Both effects were primarily driven by the yoga group, but it has to be noted that the yoga practitioners had a much higher average number of lifetime practice hours than the meditation practitioners. Moreover, the authors acknowledge the potential confounding factor that individuals with higher fluid intelligence and better functional neural network integration might be more naturally inclined to practice yoga and meditation in the first place. Hence, further studies in which both the experimental and control groups consist of naïve participants are needed.

Lastly, functional magnetic resonance imaging (fMRI) was used to investigate the neurocognitive correlates of emotion interference on executive functioning in yoga practitioners and controls with no yoga experience (Froeliger et al., 2012b). Specifically, participants performed an Affective Stroop Task (Froeliger et al., 2012c) in an event-related design, in which the individual trials were bracketed by neutral or negative emotional distractor images. Between groups there were no significant task-related behavioral differences, but there were interesting neural differences. It is known that while viewing negative stimuli, mid-frontal brain structures associated with executive cognitive control may activate to implement top-down modulation of negative affective responses (Wager et al., 2008). In yoga practitioners, prefrontal activation during negative emotion trials was greater compared to controls only during the presence of the cognitively demanding task, but not during the simple viewing of emotional stimuli *per se*. Moreover, in yoga practitioners amygdala activation to negative emotional distractors was not coupled with task-related changes in affect as measured with the Positive and Negative Affect Schedule (PANAS; Watson et al., 1988). These results suggest that yoga practitioners may be able to selectively recruit frontal executive strategies in response to emotionally salient stimuli as a function of cognitive demand. They also indicate that, as mindfulness-based practices (Hölzel et al., 2011), yoga may promote attention toward emotional stimuli without active attempts to cognitively restructure the affective experience.

## Limitations of Previous Studies on Yoga-based Practices

The relatively small body of literature on the effects of YBP that involve posture and/or movement in healthy populations reviewed above, has several methodological limitations. Below we will list some of the main points of concern, and outline a number of key factors for the systematic investigation of YBP in the future.

### Self-Selected Populations and Inappropriate Control Groups

In most of the aforementioned studies, the experimental group consisted of either advanced practitioners (David et al., 2014; Villemure et al., 2014) or individuals participating in a residential yoga program (Telles et al., 1995; Gard et al., 2012), whereas the control participants, were mostly individuals with no yoga experience. As pointed out by Gard and colleagues (Gard et al., 2014b), some of the reported physiological or behavioral differences may be driven by pre-existing characteristics of individuals who are naturally inclined to engage in YBP. Similarly, the effects of motivational and expectance factors of individuals who self-initiate interest in YBP need to be taken into account (Jensen et al., 2012). In addition, many of the studies did not include an active control group (Froeliger et al., 2012a; Fiori et al., 2014), and if they did it predominantly consisted of regular physical exercise (Oken et al., 2006; Gothe et al., 2013). This can be problematic since exercise matches YBP only in terms of overall physical demands and group participation in general. In order to broaden the evidence base for the effects of YBP it is important to conduct further studies on a larger variety of healthy and clinical populations. Studies with yoga naïve healthy participants will be particularly informative for determining the longevity and intensity of practice necessary to yield measurable effects. Individual characteristics such as body type, personality, motivation etc. that might influence a participant’s response to the practice should also be more carefully assessed. A better understanding of the mechanisms of change and the individual factors influencing responsiveness in healthy populations, will in turn allow for more precisely targeted interventions in clinical populations. More studies including random assignment to either YBP or carefully matched active control conditions are necessary. Control conditions need to be matched to YBP not only for overall physical intensity, but also for more subtle factors such as specific attentional demands.

### Use of Self-Report Outcome Measures Alone

Many of the studies assessing the effects of YBP on perceived levels of stress and emotional states have based their results on pre vs. post comparisons of self-report outcome measures alone (Wood, 1993; Malathi et al., 2000). This represents a problem when studying contemplative practices in particular, as the participants’ perception and judgment of their own skills and coping mechanisms are often altered as a result of the practices themselves. Some of the inherent cognitive biases accompanying self-report assessment of intervention outcomes have been explicitly addressed in regard to mindfulness-based practices (Grossman, 2008), and we believe that similar concerns apply to YBP. Given the commonly touted positive effect of YBP for one’s overall well-being, there is a danger of biased responses toward expecting, believing, and reporting that the intervention has worked. In future studies it will be important to more carefully develop and select outcome measures. Complementary assessment of behavioral, physiological, neural and cognitive change is necessary to obtain a comprehensive understanding of the effects. Studies combining both first person reports of experienced effects, and third person objective measurements thereof, will be particularly informative.

### Poorly Described Interventions

With few exceptions (Oken et al., 2006; David et al., 2014), the descriptions of the YBP administered to participants lack the necessary details to allow for close experimental replication of the studies. In fact, in several cases the interventions are merely referred to as a combination of asana, pranayama and relaxation techniques (Rani and Rao, 1994; Narayana, 2009). Given that there are many variations of modern YBP, this is clearly problematic. Concerted effort needs to be directed toward accurately and sufficiently describing the exact type of movement, breath and attention that are being instructed in the context of a particular intervention. This is crucial for designing appropriate control conditions, as well as for the possibility to replicate the studies.

### No Investigation of the Individual Components of YBP

YBP are inherently multifaceted in nature, typically involving a combination of specific postures or movement sequences, specialized use of the breath, and various techniques to promote sustained attention (Gard et al., 2014b). So far, little if any research has attempted to deconstruct the role of these different component parts. Hence, it remains unclear to which extent the movement, the breath or the attentional focus are driving the effect on the observed changes in physiology, emotional states and cognition, and whether the effect of these components is synergistic in nature (Payne and Crane-Godreau, 2013). To shed further light on the factors that drive the effect of YBP, an important avenue for future research studies is to attempt to disentangle the respective contribution of the individual component parts in more detail. Investigations in which the type, amount or intensity of either movement, breath or attention are carefully manipulated, while the other components are kept constant, will be particularly informative in this regard. Such studies will allow the investigation of which specific outcome measures are primarily impacted by each of the components.

### Lack of Hypotheses About the Specific Mechanisms of Change Underlying the Reported Effects of YBP

While some studies outline hypotheses about neurophysiological and neurocognitive mechanisms underlying the results (Froeliger et al., 2012b; Gard et al., 2012; Villemure et al., 2014), these hypotheses mostly refer to general mechanisms known to underlie the effect of mindfulness-based practices. Other studies (Rani and Rao, 1994; Kamei et al., 2000; Malathi et al., 2000) don’t provide any detailed mechanistic hypotheses at all. Recently proposed theoretical frameworks about the effects of YBP are an encouraging step forward (Gard et al., 2014a; Henje Blom et al., 2014a), but the proposed mechanisms have yet to be systematically tested in healthy populations. Future studies on YBP should be driven by specific hypotheses about the mechanisms of change that are being targeted. Hypotheses about the interaction between bottom-up physiological and top-down cognitive processes that can be systematically tested will be of particular relevance. Testing of such hypotheses will require multidisciplinary approaches across the domains of physiology, neuroscience and psychology.

## The Main Components of Modern Yoga-based Practices

We will now outline and provide a detailed description of three fundamental components of modern YBP, namely movement, breath and attention. A deconstruction of these components is very important when designing and describing interventions aimed at better understanding their respective effects.

### Movement

What constitutes “movement” in the context of movement-based contemplative practices is not necessarily easy to define, as it can range from large overt motion, to small subtle motion, and often even to purely internal or imagined motion (Schmalzl et al., 2014). Here we refer to movement in the sense of the overt execution of specific physical postures or movement sequences. This type of practice is very prominent in modern yoga, and there are many different schools that differ in the type of movement they entail. Some practices, such as Ashtanga Vinyasa Yoga (Jois, 1999), are characterized by quite intense and continuous physical motion with a focus on creating a “flow” of movement by linking one posture to the next. In other practices, such as Iyengar Yoga (Iyengar, 1966) or other forms of Hatha Yoga (Akers, 2002), the movement is less dynamic and the focus is on holding individual postures for a longer period of time. Independently of its historical origin (Singleton, 2010), the movement aspect of modern YBP has potentially important therapeutic implications such as physical/physiological benefits, fine-tuning of interoceptive and proprioceptive awareness, and providing a context for training attention. Below we will outline the types and characteristics of movement employed in modern YBP that sets it apart from more common forms of physical exercise.

#### Postures

Both static and dynamic forms of YBP involve the execution of specific physical postures. The postures are overall designed to increase range of motion, strength and flexibility. They are mostly taught using precise alignment cues, and depending on the type of practice they can be held for just a few breaths or up to a few minutes. There are innumerable individual postures and variations thereof that can be descriptively categorized into standing, seated, and supine postures, or into forward folds, backbends, hip-openers, twists and inversions.

#### Movement Sequences

When YBP involve dynamic movement sequences, these are often performed in a slow, rhythmic and symmetric fashion that is synchronized with the breath so as to create a flow from one pose to the next. The joint load is mostly kept at submaximal levels, which has been suggested to be beneficial for bone remodeling and osteogenesis (Omkar et al., 2011).

#### Interior Muscle Activations

Another common aspect of current posture practice across many yoga styles is the use of a static and soft contraction of interior muscle groups at the level of the pelvic floor, the lower abdomen and the throat. The activation of these muscle groups aids breathing practices and facilitates the maintenance of a strong core musculature while moving through the postures. Within some of the traditional yoga systems these muscle activations are referred to as “bandhas”, and described as seals that can direct the flow of prana in the body.

#### Coordinated Movement of Moderate Intensity

YBP are mostly performed in a slow and controlled manner and require balance, coordination, as well as a constant tracking of the body’s position in space. Postural alignment, fluidity and fine-tuning of the movement are emphasized. In contrast to many forms of physical exercise that tend to increase activity in the sympathetic nervous system, it has been proposed that the level of intensity employed in YBP is likely to increase parasympathetic tone and consequently promote down-regulation of stress levels (Larkey et al., 2009).

#### Expansion of the Range of Motion

In many yoga styles, posture practice is aimed at expanding range of motion. A common belief is that we “hold” tension in our muscular system, and that the accumulation of both physical and emotional stress over time manifests as stiffness and blockages in our muscles, joints and connective tissue. A putative aim of posture practice is therefore to release this tension by directing attention to the physical limitation, while directly moving towards and breathing into it. One may hypothesize that the reduction of experiential avoidance that comes with this type of practice may be generalized to other behaviors and increase psychological flexibility. This approach differentiates many YBP from other forms of movement-based contemplative practices such as qigong, which hold the view that approaching the limits of ones range of motion might actually increase tension and resistance, and consequently impede the flow of “qi” or energy (Cohen, 1999).

#### Tracking of Bodily Sensations

A fundamental aspect of YBP is paying attention to interoceptive, proprioceptive, kinesthetic and spatial sensations, and using that information to adjust and fine-tune one’s movements. Interoceptive awareness refers to the awareness of internal bodily states and sensations, including heart rate, respiration, as well as several autonomic nervous system responses related to emotional states (Cameron, 2001). The importance of interoceptive awareness is manifold. In his theory of somatic markers, Damasio (Damasio, 1996) posits that interoceptive awareness is essential for most affective, cognitive as well as interpersonal processes. In fact, it underlies one’s sense of affective and autonomic state, which is in turn fundamental for relating to the outer world (Damasio, 2000). The processing of bodily sensations is also a key for our sense of bodily self, which originates through the integration of interoceptive, proprioceptive, kinesthetic, tactile and spatial information (Ehrsson, 2007; Haselager et al., 2011; Ionta et al., 2011). Studies on tai chi as well as meditation techniques have shown that these practices can alter tactile acuity (Kerr et al., 2008), interoceptive accuracy (Fox et al., 2012), and in fact the cortical representation of interoceptive attention by impacting connectivity between the posterior and anterior insula (Farb et al., 2013). Recent evidence for increased pain tolerance in advanced yoga practitioners (Villemure et al., 2014), putatively mediated by adaptive insular changes and increased interoceptive processing, suggests that YBP may also promote similar effects.

#### Intent of Obtaining a State of Eutony

Lastly, yoga-based movement is practiced with the intent of obtaining a balanced muscle tone that allows the movement to feel stable and well rooted, yet light and effortless. While individual postures or parts of the practice may be characterized by a hypertonic (e.g., arm balances that require a high level of muscle tension) or hypotonic (e.g., a supine relaxation pose) state, the overall aim of the practice is to create a state of eutony or “well-balanced tension” (Alexander, 1985).

### Breath

It is beyond the scope of this paper to explore the multitude of breathing practices found within different yoga traditions. We will instead simply outline the different ways in which breath awareness and conscious breath regulation are emphasized in the context of modern practices. In dynamic YBP there is often a focus on precisely coordinating the movements with the breath. In other types of YBP, the postures are sometimes maintained during breath retention, or the breath serves merely as an object of attention as in many meditation practices. The conscious practice of altering breathing patterns may have a number of different effects depending on their characteristics (Brown and Gerbarg, 2005a). For instance, slow and rhythmic breathing is said to promote a shift to parasympathetic dominance via vagal afferent stimulation with consequent stress reduction (Sovik, 2000), whereas more forceful breathing practices may promote sympathetic activation (Beauchaine, 2001).

In some systems, e.g., Ashtanga Vinyasa Yoga (Jois, 1999), each posture and movement sequence is coupled to a specific breathing rhythm so that specific movements help enhance the breath (and *vice versa*). For example, expansive movements (e.g., chest openers) facilitate inhalation, whereas contractive movements (e.g., forward folds) facilitate exhalation. In other types of practices the focus is simply on cultivating an even rhythm of inhalations and exhalations, with no specific emphasis on linking movement with breath. Practicing breath awareness and conscious breath regulation in the context of movement has the potential of facilitating the use of supportive breathing patterns in everyday life situations. The breath can also be harnessed as a tool to direct attention to specific body parts while holding a posture or performing a movement, and to consequently increase interoceptive and proprioceptive awareness. In addition, the breath can be used to “turn towards” unpleasant or stressful sensations that arise in the context of the physical practice, and to “breathe into them” rather than avoid or fight them.

One example of an often-used breathing technique in modern YBP is “ujjayi breath” (Brown and Gerbarg, 2005a). It is a deep, slow and rhythmic breath in which involves visualizing the inhalations starting from the lower belly, continuing in a wave through the ribcage, upper chest and throat, and subsequently the exhalations in the opposite order. Both inhalations and exhalations are performed through the nostrils with concurrent narrowing of the throat passage at the level of the glottis, which creates a soft and soothing sound. When ujjayi breath is performed during dynamic movement practice, inhalations and exhalations are ideally of equal duration. The duration is controlled by the diaphragm, and can gradually extend with practice. It is emphasized not to force or hold the breath, to avoid muscular tension.

Western science on respiratory physiology supports the view of some Eastern yoga traditions that emotional states are expressed in breathing patterns, and subsequently that voluntary change of the breathing patterns can alter emotional states and influence wellbeing (Boiten et al., 1994; Brown and Gerbarg, 2005a; Henje Blom et al., 2014b). In fact, a typical autonomic reaction to stressful situations is rapid thoracic breathing, which in turn leads to hyperventilation, altered tidal volume and hypocapnia (Laffey and Kavanagh, 2002). These symptoms are frequently observed as chronic manifestations in individuals with anxiety and depressive disorders (Meuret et al., 2004), and may be alleviated by the types of breathing techniques described above (Brown and Gerbarg, 2005b).

### Attention

Regulation of attention is a central aspect of most contemplative practices, and YBP are no exception. We will first outline commonly used categories of attention from a cognitive science perspective. Subsequently, we will describe some more specific types of attentional focus and how they relate to YBP.

#### Commonly Used Categories of Attention

Though a variety of attention theories exist, one predominant characterization divides attentional processing into three main functional branches—alerting attention, orienting attention, and executive functioning (Posner and Boies, 1971; Fan et al., 2002, 2003). Each of these has been linked to distinct neural networks (Petersen and Posner, 2012). Alerting attention, also referred to as vigilance, sustained attention, or task-specific phasic alertness, refers to a general ability to track, and preparedness to respond to, environmental or task related stimuli (Raz and Buhle, 2006). In YBP, alerting attention is primarily recruited for tracking bodily sensations, which we will outline in detail below. Orienting attention involves active scanning of the environment or an array of stimuli, and subsequent orientation toward, or selection of, a specific target for the execution of a behavior or task. In YBP, orienting attention supports fine-tuning of neuromuscular feedback processing, and the consequent efficiency of muscle engagement for the execution of physical postures, movement sequences, and breathing techniques. Executive attention refers to the ability to selectively pay attention to relevant stimuli in our environment, while contemporarily inhibiting irrelevant information. In YBP, executive functioning is used to maintain attention on present-moment physical and mental states, while simultaneously withholding attention from irrelevant distractions.

#### Attention to Bodily Sensations

One of the principal defining characteristics of YBP, akin to other forms of movement-based contemplative practices (Schmalzl et al., 2014), is the emphasis on becoming increasingly attentive to bodily sensations and sensory experiences. This includes the cultivation of interoceptive, proprioceptive, kinesthetic and spatial awareness, while primarily paying attention to the movement and breath components of the practice described above. Such direct focus as a goal in its own right differentiates YBP from most other types of conventional exercise, where the goal is often external in nature (e.g., hitting a target, performing a specific action, reaching a destination etc.).

High degrees of body awareness have been associated with both negative and positive effects (Mehling et al., 2012). On the one hand, excessive focus on bodily sensations can be an indication of somatization, anxiety and even depressive symptoms. In this case, enhanced body awareness often takes on the form of hyper vigilance, rumination and over interpretation of bodily signals. On the other hand, there is increasing consensus that body awareness and the ability to detect subtle bodily cues can be beneficial for health and self-regulation. In this case, enhanced body awareness reflects an increased ability to observe bodily signals as such without getting caught up in them (Baas et al., 2004). One goal of training body awareness for therapeutic purposes is therefore to increase proprioceptive and interoceptive awareness, while reducing self-evaluative processes (Watkins and Teasdale, 2004). On a neural level this has been proposed to correlate with a shift from predominantly medial prefrontal activation, to increased activation of the thalamus, the insula and primary sensory regions (Farb et al., 2012). We hypothesize that compared to seated contemplative practices, the added movement component of YBP may increase the intensity of interoceptive and proprioceptive signals, and subsequently facilitate their processing and integration. Hence, YBP may offer a potentially even more efficient method for cultivating bodily awareness as well as general attentional skills.

#### Focused Attention vs. Open Monitoring

Contemplative practices use a variety of techniques to train focus of attention (Jha et al., 2007; Lutz et al., 2008; Manna et al., 2010). Meditation practices are often classified as engaging predominantly focused attention (FA) or open monitoring (OM) techniques (Lutz et al., 2008), and it is not yet well-defined where YBP fall on that spectrum. In short, FA techniques involve directing and sustaining attention on a single selected object (e.g., the sensation of the breath), whereas OM techniques emphasize non-reactive metacognitive monitoring of perceived sensory, emotional or cognitive events that may arise from moment to moment during one’s practice.

In the context of mindfulness-based practices, it has been argued that while beginners tend to primarily engage in FA, advanced practitioners gravitate more toward an OM approach (Lutz et al., 2008). We propose that individuals engaging in YBP are also likely to gradually transition from a FA to a more OM attentional orientation. Novice practitioners may only be able to allocate their attention to one single element of the practice at the time, but as their practice advances they are likely to become increasingly skilled at simultaneously monitoring movement, breath, and any concomitant interoceptive and exteroceptive sensations that may arise. Hence, we hypothesize that YBP, at least in more advanced practitioners, primarily engage an OM type of attention.

#### Metacognitive Awareness

Metacognition can be broadly defined as the conscious and mostly intentional monitoring of our own mental processes and behaviors (Teasdale, 1999). The action of “stepping back” to observe ones own inner sensations and thoughts is central to most contemplative practices including YBP. It is also an aspect that differentiates YBP from many common forms of exercise, which are often practiced without a primary goal of paying attention to bodily or mental states (e.g., running on a treadmill while listening to music).

To our knowledge there are no studies that have directly investigated the effect of YBP on metacognition as such. Hence, we will refer to some insights from the literature on mindfulness-based practices, which may also apply to YBP. It has recently been proposed (Fox and Christoff, 2014), that alongside creative thought and lucid dreaming, mindfulness-based practices represent a third quite unique scenario of interplay between metacognition and so-called mind-wandering (MW), which refers to spontaneous and undirected thought processes that mostly occur without our volition (Kane et al., 2007). With reference to the FA vs. OM distinction addressed above, there are two broad possibilities for the co-existence of MW and metacognition. In the context of FA, metacognition has the “suppressive” function of noticing drifts of attention from a selected object, and subsequently of redirecting attention towards it. In the context of OM, metacognition has the more “integrated” function of monitoring one’s stream of thought, while attempting to maintain detachment and refrain from any cognitive elaboration or judgment.

One proposed benefit of a consistent and non-judgmental metacognitive monitoring of sensations and spontaneous thought processes is reduced negative self-referential rumination (Deyo et al., 2009). Repeated practice of simply monitoring one’s sensations and thoughts without trying to interpret or judge them, is said to lead to a gradual lessening of one’s identification with those thoughts. Negative self-referential thoughts are progressively seen as mere temporary mental events, and therefore not as negative self-defining reifications. A second proposed positive effect is an enhanced sense of equanimity (Desbordes et al., 2015), a non-judgmental metacognitive awareness of the broad spectrum of sensations and thoughts that may arise at any given point in time. In the context of YBP, we propose that this process is first primarily applied to bodily sensations and proprioceptive feedback related to the movement and breath, and subsequently also to arising emotions and thoughts. Preliminary support for this view comes from the finding that yoga practitioners seem to exhibit increased pain tolerance, putatively mediated by acquired tendencies to simply attend to the sensation, observe the pain without reacting, and accept the associated experience (Villemure et al., 2014). On a similar note, yoga practitioners were found to exhibit less top-down cognitive control when viewing emotional stimuli, suggesting less engagement in active attempts to cognitively restructure the affective experience (Froeliger et al., 2012b).

#### Use of the Gaze as a Tool for Training Attentional Focus

Some YBP use gaze as a tool for training attention and inducing a calm state of mind (Hedstrom, 1991). A few texts of YBP describe details of “eye exercises” (Satchidananda, 1970) including gazing techniques in which the eyes are held in a particular position (e.g., upward, inward or downward). These exercises are recommended to “aid powers of concentration” (Schwendimann, 1961), and prevent one’s attention from being distracted (Bahm, 1965). YBP often involve explicit instructions to avoid eye movements to potentially distracting stimuli in the visual environment, and to instead cultivate a controlled gaze towards specific body parts. There is typically an emphasis on performing eye movements consciously and slowly, and on coordinating them with the breath as well as specific physical postures. Lastly, it has been proposed that gaze directed within the upper visual field promotes more allocentric referential processing relative to gaze directed within the lower visual field (Sdoia et al., 2004). Since YBP employ both, they may promote the dynamic integration of allocentric and egocentric reference frames, which may in turn facilitate the ability to monitor the visual environment with less personal bias (Austin, 2009).

## Neurocircuitry and Physiological Processes Hypothesized to be Implicated in Yoga-based Practices

**Figure 2:**
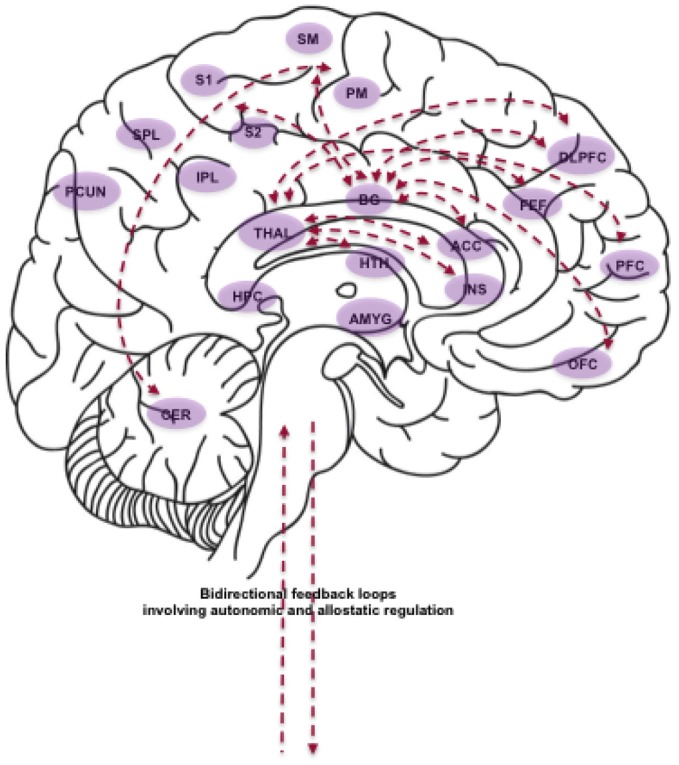
**Schematic depiction of some of the brain areas, neural circuits and physiological processes proposed to be affected by YBP. Abbreviations (in alphabetical order)**: ACC (Anterior Cingulate Cortex); AMYG (Amygdala); BG (Basal Ganglia); CER (Cerebellum); DLPFC (Dorsolateral Prefrontal Cortex); FEF (Frontal Eye Fields); HPC (Hippocampus); HTH (Hypothalamus); INS (Insula); IPL (Inferior Parietal Lobule); OFC (Orbitofrontal Cortex); PCUN (Precuneus); PFC (Prefrontal Cortex); PM (Premotor Cortex); S1 (Primary Somatosensory Cortex); S2 (Secondary Somatosensory Cortex); SM (Supplementary Motor Cortex); SPL (Superior Parietal Lobule); THAL (Thalamus). **The role of BG and cerebellar circuits**: BG circuits involving PM and SM support the coordination of complex movements. BG circuits involving the DLPFC, OFC and ACC support executive functioning and procedural learning implicated in the planning and learning of motor sequences. BG circuits involving the DLPFC additionally support working memory required for the execution of motor sequences. BG circuits involving S1, S2, DLPFC, and ACC support somatosensory processing as well as the perception of noxious stimuli. BG circuits involving FEF support voluntary eye movements and gaze control. Lastly, CER circuits involving PM and SM support motor coordination and action execution. **Brain areas and neural circuits supporting body-focused attention and interoceptive awareness**: The INS is the key neural structure for the processing of both exteroceptive (somatic) and interoceptive (physiological) information for bodily awareness and autonomic regulation. The ACC supports the processing of noxious stimuli as well as error detection and conflict monitoring. S1 and S2 are the core regions for the processing of tactile and proprioceptive sensations. The PCUN supports higher-order body awareness, self-related processing and attentional shifting. The IPL and SPL are involved in the computation of body size and shape, and in the integration of multimodal spatial information in body-centered coordinates. The PFC supports executive control and self-referential processing. **Integration of bottom-up and top-down processes**: The breath employed in YBP putatively promotes synchronization of cortical areas via stimulation of THAL nuclei, with a consequent positive impact on alertness and executive functioning. In addition, slow and rhythmic breathing is known to promote vagal tone and in turn reduce allostatic load. The THAL mediates vagal afferent information to the INS, ACC and PFC, which are all involved in self-regulatory processes. The AMYG supports fear-detection, and consequent modulation of autonomic states. The HPC contains stress hormone receptors that can influence the evaluation and memory of stress-related events.

Having deconstructed the components of movement, breath and attention, we will now outline the main implied neurocircuitry and physiological processes (Figure 2). Our aim is to inform specific hypotheses regarding the mechanisms of change affected by YBP.

### Neurocircuitry Implicated in Movement-Related Aspects of YBP

Many of the movement-related aspects of YBP engage basal ganglia (BG) and cerebellar circuits. The BG comprise a group of subcortical gray matter nuclei located at the base of the forebrain, including the striatum (putamen, caudate nucleus and nucleus accumbens), the globus pallidus, the subthalamic nucleus, and the substantia nigra (Martin, 2003). The BG are connected to the cortex and the cerebellum via a series of semi-independent loops (McHaffie et al., 2005). Each of these loops originates from a specific cortical region, passes through functionally corresponding portions of the BG, and returns to the same cortical area via the thalamus (Alexander, 1994). The major input nuclei of the BG are the putamen and caudate nucleus, which receive afferent information from the cortex (cortico-striatal projections) as well as the intralaminar nucleus of the thalamus (thalamo-striatal projections). The majority of top-down cortico-striatal projections come from five regions of the frontal lobes—the premotor and supplementary motor areas, the frontal and supplemental eye fields, the orbital frontal cortex, the dorsolateral prefrontal cortex, and the anterior cingulate. The major output nuclei of the BG are the globus pallidus and the pars reticulate of the substantia nigra, which project in turn to the thalamus and back to the cortex—primarily to premotor and motor cortices as well as to prefrontal areas (Arsalidou et al., 2013).

As mentioned earlier, YBP involve the execution of postures and movement sequences that require a high degree of coordination. The BG support this type of bodily movement via loops with cortico-striatal projections from premotor and supplementary motor areas (Arsalidou et al., 2013). These loops are part of the extrapyramidal motor system, which is responsible for the modulation of neural impulses that originate in the cerebral cortex. Such modulations are involved in the initiation and selective activation of certain movements while suppressing others, setting the rate and force of movements, and coordinating movements. Hence, a central function of these loops, which operate in conjunction with parallel loops involving the cerebellum, is the selection and triggering of well-coordinated voluntary movements (DeLong and Georgopoulos, 2011).

Movement in the context of YBP also involves executive functioning and procedural learning, which are implicated in the planning and learning of motor sequences as well as in decision making according to neuromuscular feedback in the context of postural adjustments. The BG support executive functioning via loops with cortico-striatal projections from the dorsolateral prefrontal cortex, the orbitofrontal cortex, and the anterior cingulate cortex (ACC; Arsalidou et al., 2013). Such executive processes include planning, problem solving, set-shifting and decision making, which are all crucial for procedural learning (Monchi et al., 2006). BG involvement in procedural learning has in fact been shown across several modalities (including kinesthetic/vestibular, visual, olfactory, and auditory), and been found to generalize across mammalian species (Packard, 2009).

The execution of postures and movement sequences in YBP also relies on working memory, as it requires the ability to hold in mind instructions and consequently select very specific sequential motor actions. The BG support working memory via loops with cortico-striatal projections from the dorsolateral prefrontal cortex (Arsalidou et al., 2013). In humans, these loops BG seem to be particularly involved in motor habit learning (DeLong and Georgopoulos, 2011). An important function of working memory in this context is to predict a forthcoming event so that the motor system can prepare for action. That is, based on information stored in working memory, and through mechanisms of inhibition and dis-inhibition, the BG are responsible for “opening the gate” for specific motor actions in a predictive manner (Hikosaka et al., 2000).

The fact that a large majority of the initial studies investigating the functional role of the BG were based on patients with motor dysfunction such as Parkinson’s Disease (Chenery et al., 2008) or Huntington’s Disease (Paulsen, 2009), led to the traditional view that the BG are primarily associated with motor functions. However, subsequent studies have shown that the BG are also implicated in cognitive disorders such as attention deficit hyperactivity disorder (ADHD; Knutson and Gibbs, 2007) and obsessive compulsive disorder (OCD; Huyser et al., 2009). Moreover, converging evidence from studies in healthy populations suggests that BG are involved in a series of more complex functions including somatosensation, higher order cognitive functions, and even social behavior (Arsalidou et al., 2013), all of which seem to rely on at least partially differential cortico-BG feedback loops. The same is true for the cerebellum, which has long been known for its involvement in motor coordination, but more recently also recognized for its involvement in cognitive functioning and emotional processing (Schmahmann and Sherman, 1998; Strata et al., 2011; Baumann and Mattingley, 2012). Action execution and task performance are primarily supported by motor-cerebellar circuits, whereas cognitive functioning and emotional processing are primarily supported by prefrontal-parietal-cerebellar circuits (Balsters et al., 2014). We hypothesize that YBP may promote increased connectivity within, and dynamic shifting between, motor, cognitive and emotional neurocircuitry of both the BG and the cerebellum, with potential beneficial effects for mind-body integration and self-regulation. Supporting evidence for this hypothesis comes from a recent neuroimaging study documenting more widespread functional connectivity within BG cortico-thalamic feedback loops in yoga and meditation practitioners compared to controls (Gard et al., 2015).

### Physiological Effects of the Breath Employed in YBP

It has been shown that slowing down the breathing rate to about six breaths per minute with matched inhalations and exhalations decreases chemoreflex sensitivity—i.e., the naturally occurring change in breathing rate in response to changes in the concentration of oxygen and carbon dioxide in the blood (Spicuzza et al., 2000). Application of this specific breathing rhythm also decreases oxidative stress—i.e., an imbalance between the production of reactive oxygen species and antioxidant defenses (Sharma et al., 2003). In addition, paced breathing increases the release of prolactin and oxytocin release which can promote feelings of calmness and social bonding (Torner et al., 2002).

The previously described ujjayi breath involves contraction of laryngeal muscles, which creates an increase of airway resistance hypothesized to stimulate somatosensory vagal afferents to the brain (Brown and Gerbarg, 2005a), and in turn promote improved autonomic regulation (Calabrese et al., 2000). Furthermore, breath employed in YBP may promote synchronization of cortical areas via stimulation of thalamic nuclei, with a consequent positive impact on alertness and executive functioning (Calabrese et al., 2000; Tsigos and Chrousos, 2002).

Lastly, it has been shown that fluctuations of depth and rate of breathing during fMRI scanning correlate with blood-oxygen-level dependent (BOLD) signal changes in regions with high blood volume (Birn et al., 2006). Interestingly, these changes have been found to largely overlap with the so-called default mode network (DMN) consisting of brain regions that are active during wakeful rest (Raichle et al., 2001). Other regions showing the strongest breath-related signal changes include the cerebellum, BG (putamen and caudate nucleus), insula, ACC, orbitofrontal cortex, dorsolateral and ventrolateral prefrontal cortex, and the supplementary motor area (Chang and Glover, 2009).

### Neural Correlates of Attention Regulation in YBP

#### Body-Focused Attention and Interoceptive Awareness

Most of what we know to date about the neural correlates of body-focused attention in contemplative practices is based on studies of body-centered meditation and mindfulness-based techniques with a limited active movement component. According to a recent meta-analysis (Fox et al., 2014), gray matter regions that have been found to undergo structural changes associated with meditation practice include the insular cortex, primary and secondary sensorimotor cortices, and the anterior precuneus. The insula is a key neural substrate for interoceptive awareness (Critchley, 2005; Craig, 2009). Information about interoceptive signals is first transmitted via the brainstem and the thalamus first to the posterior insula, which is the primary interoceptive cortex. Subsequently, it is transmitted to the anterior insula, where the information is integrated with other contextual information that make it accessible to consciousness (Damasio and Carvalho, 2013). Primary and secondary somatosensory cortices represent the main locus for the processing of tactile and proprioceptive sensations (Venkatesan et al., 2014). The precuneus in contrast has been suggested to play an important role for higher-order body awareness as well as more generally for self-related processing and attentional shifting (Cavanna and Trimble, 2006). In terms of white matter pathways relevant to body-focused attention, structural changes have been reported in the superior longitudinal fasciculus (Fox et al., 2014). It consists of rostro-caudal fiber pathways that connect dorsal temporo-parietal regions with prefrontal regions (Makris et al., 2005). Hence, it can be assumed to play an important role in connecting parietal body awareness regions and prefrontal executive regions.

A meta-analysis of functional brain changes associated with meditation practices (Tomasino et al., 2013), also revealed changes in a number of areas involved in the processing of bodily signals, likely reflecting the body-focused attention applied in these practices. Specifically, consistent changes in activation have been reported in parietal areas involved in spatial and somatosensory processing (the superior and inferior parietal lobule), the right supramarginal gyrus, and again the insular cortex. The superior parietal lobule is involved in integrating multimodal spatial information in body-centered coordinates (Felician et al., 2004). The inferior parietal lobule is involved in the computation of the size and shape of the body and body parts (Ehrsson et al., 2005). The supramarginal gyrus has been found to be activated during disembodiment and altered integration of multisensory information (Blanke and Mohr, 2005). And lastly, as mentioned previously, the insular cortex is known for its role in processing both exteroceptive (somatic) and interoceptive (physiological) information that is crucial for bodily awareness and autonomic regulation (Critchley, 2005).

There is preliminary evidence that YBP may also be associated with structural changes in brain areas involved the processing of bodily sensations. In fact, Villemure and colleagues (Villemure et al., 2014) found that yoga practitioners had increased GMV in the insula, cingulate cortex, medial prefrontal cortex, and inferior and superior parietal lobule, as well as increased intra-insular white matter connectivity. Similarly, Froeliger and colleagues (Froeliger et al., 2012a) also found increased GMV in the insula and cerebellar regions of yoga practitioners compared to controls. While these findings are certainly of interest, it is important to keep in mind that it is not yet known to which extent they reflect changes driven by aspects specific to YBP, as opposed to changes driven by more general aspects of bodily attention also employed in mindfulness-based techniques. Studies directly comparing movement-based yoga-practices and standard mindfulness-based practices will be crucial for addressing this point.

Further expanding on the neural correlates of increased body awareness, Kerr and colleagues (Kerr et al., 2013) outlined a theoretical framework proposing that body-focused attention elicits changes in brain dynamics that enhance signal-to-noise ratio in attentional processing across different modalities. Specifically, they propose that somatically focused practice enhances attentional control of the 7–14 Hz alpha rhythm, which is said to be crucial for regulating input and signal-to-noise ratio not only for sensory cortices but across the neocortex (Kerr et al., 2011). The concrete and tangible nature of somatic information and feedback is thought to represent an efficient “tool” for learning how to modulate the alpha rhythm, so that with sustained practice the flow of information is then more efficiently filtered and prioritized throughout the brain. The view that localized somatosensory alpha modulation training can lead to a more general increase of attentional control, is consistent with studies on body-focused mindfulness practices showing long-term changes in prefrontal cortex activation (Davidson et al., 2003; Farb et al., 2012), or enhanced performance in tests of visual selective attention (Jha et al., 2007; Jensen et al., 2012). In addition, the sensory alpha modulation framework is corroborated by the proposal that the transition from FA to a more OM type of attention by experienced meditation practitioners, may be partly promoted because of improved control over alpha rhythm phase dynamics (Mathewson et al., 2011). The extent to which these findings of functional brain changes and the consequent general attentional enhancement associated with mindfulness-based practices will also be found in YBP, remains a fundamental question to be addressed by future research studies.

Lastly, we would like to briefly address the role of the BG in body awareness. The BG are involved in somatosensory processing and the perception of noxious stimuli via loops with cortico-striatal projections from somatosensory areas, the dorsolateral prefrontal cortex and ACC (Arsalidou et al., 2013). Many BG neurons are responsive to somatosensory stimulation, and particularly to nociceptive stimulation. In fact, evidence from neurophysiological, clinical and behavior experiments suggests that the BG are uniquely involved in the complex function of integrating motor, emotional, autonomic and cognitive responses to pain (Borsook et al., 2010). Such processing is particularly relevant for YBP, which often involve challenging physical and emotional sensations.

#### Mind-Wandering and Metacognition

In the previous section of our paper we proposed the view that YBP may primarily employ an OM type of attention, with a constant interplay of MW and metacognitive awareness. MW is said to predominantly engage regions of the DMN (Raichle et al., 2001), with self-referential spontaneous thoughts being reflected by activation of the posterior cingulate cortex (PCC) and the anterior medial prefrontal cortex in particular (Andrews-Hanna, 2012). Metacognition, on the other hand, is said to predominantly engage higher order prefrontal regions (Fleming and Dolan, 2012) including the anterior and dorsolateral prefrontal cortices, as well as the ACC and the anterior insula, which is proposed to subserve meta-awareness of internal bodily states (Critchley, 2005).

Neuroimaging studies of mindfulness-based practices have provided evidence for alteration of both DMN (Ives-Deliperi et al., 2011) and metacognitive (Manna et al., 2010) brain regions, indicating that contemplative practitioners may indeed consistently engage both. We speculate that the same is true for YBP. As mentioned earlier, the practice of non-judgmental metacognitive monitoring of sensations and spontaneous thought processes is likely to promote increased emotional awareness, non-reactivity and equanimity. Cultivating emotional awareness implies involvement of brain circuitry implicated in emotional regulation such as the limbic system, the ACC and prefrontal regions (Farb et al., 2012). In addition, the BG support emotion processing via loops with cortico-striatal projections from the dorsolateral prefrontal cortex and ACC (Arsalidou et al., 2013).

#### Gaze Training

Studies investigating the relationship between gaze and self-regulation of alpha waves in the brain, provide relevant information about the neural processes that are affected when gaze is used to train attention. It is known that the presence of alpha rhythm in occipital/visual brain regions is associated with a state of relaxed wakefulness (Haegens et al., 2014). While alpha waves typically occur with closed eyes, individuals can be trained to induce alpha waves with eyes open as long as the attention is “turned inward” (Green et al., 1979). It is suggested that alpha production in this case is related to a defocus and relaxation of ocular convergence, a technique that is very similar to a yogic eye posture known as “bhrumadhya dhrishti” (Bahm, 1965).

Controlled gaze towards specific body parts, and concomitant avoidance of eye movements towards distracting stimuli, has also been associated with specific neural correlates. fMRI data indicate that inhibiting saccades and redirecting gaze toward a target engages a frontal oculomotor network including the medial frontal cortex, frontal and supplementary eye fields, and the striatum, known to be involved in action inhibition and performance monitoring (Thakkar et al., 2014). Furthermore, evidence from ERP studies suggests that gazing at a body part enhances tactile acuity (Forster and Eimer, 2005), spatial attention, (Gherri and Forster, 2014), and more generally activation of fronto-parietal networks representing peripersonal space (Gillmeister and Forster, 2010).

Finally, important neural structures for gaze control are the BG. Specifically, the BG are involved in voluntary eye movements via loops with cortico-striatal projections from frontal and supplemental eye fields via the superior culliculus (Arsalidou et al., 2013). These loops are implicated in controlling saccadic eye movements, in particular in preventing distracting visual input from triggering unwanted saccadic eye movements, as well as smooth pursuit (Hikosaka et al., 2000). Given that spatial orienting through eye movements is known to be associated with the orienting of attention (Schneider and Deubel, 2002), this function of the BG is likely to play an important role in fostering the attentional control applied in YBP.

## Hypothesized Effects of Yoga-based Practices on the Regulation of Allostatic Load and the Integration of Bottom-up and Top-down Processes

We will now address two further possible aspects that may underlie the mechanisms of change promoted by YBP, namely the regulation of allostatic load and the integration of bottom-up and top-down processes (Figure 2).

### Regulation of Allostatic Load

The concept of allostasis refers to the ability of an organism to maintain stability/homeostasis through change by actively adjusting to both predictable and unpredictable events (McEwen and Wingfield, 2003). In humans, primary mediators of allostasis include, but are not restricted to, hormones of the hypothalamo–pituitary–adrenal (HPA) axis (e.g., cortisol), excitatory catecholamines (e.g., adrenaline), and immunomodulatory cytokines (e.g., interleukins). An imbalance of these primary mediators results in allostatic state, and cumulative effects of sustained allostatic state over time in turn results in allostatic load (Juster et al., 2010). It is important to note that while most primary mediators of allostatic load can have protective effects in the short run, the physiological integrity of the organism is compromised if the allostatic load is sustained over time (McEwen, 1998).

One of the key components for the regulation of allostatic load in humans is the vagus nerve, the 10th of the cranial nerves. Its axons emerge from and converge onto four different brainstem nuclei, and it regulates several visceral organs, as well as striated muscles of the face, head and neck (Porges, 1995). The majority (80–90%) of the vagal nerve fibers are afferent, thus communicating peripheral information about bodily states to the brain (Berthoud and Neuhuber, 2000). The function of the vagus nerve has evolved phylogenetically, and in mammals the vagus system is primarily involved in mediating stress responses by regulating CO and influencing engagement/disengagement with the environment (Porges, 2001). In fact, physical, affective as well as cognitive and social processes have all been shown to be associated with vagally mediated cardiac function (Porges, 2007). It has been proposed that vagal tone can be assessed by measuring the variability of the inter-beat intervals of the heart, i.e., HRV (Porges, 2001), and that vagal tone is especially mirrored by the HRV within the frequency of normal respiration rate, i.e., respiratory sinus arrhythmia (RSA; Calabrese et al., 2000). Furthermore, it as been hypothesized that there is an interaction between breathing frequency and HRV as well as arterial baroreflex sensitivity, with slower breathing rates promoting an increase of both these indices (Bernardi et al., 2001). However, the complex interplay between heart rate and vagal sensory input call for more careful analyses (Berntson et al., 2007).

We speculate that YBP are intrinsically tailored to promote vagal tone and facilitate a decrease of allostatic load in several ways. First, there is the direct parasympathetic effect of slow and rhythmic breathing with increased airway resistance known to increase HRV (Brown and Gerbarg, 2005a). Second, many of the postures employed in YBP enhance the depth of the breath (e.g., active expansions/contractions of the rib cage during back/forward bends), strengthening core diaphragmatic muscles and enhancing baroreceptor sensitivity (Strongoli et al., 2010). Third, most postures emphasize abdominal tone through the application of interior muscle activation, which additionally promotes peripheral vagal stimulation and afference (Ritter et al., 1992). Lastly, on a more indirect level, the practice of maintaining a calm breathing rhythm during the physical, mental an emotional challenges of the postures and movement sequences, represents an opportunity to apply non-reactive awareness and cultivate a state of equilibrium in the face of stress. Hence, YBP represent an effective way of developing strategies for dealing with stressful experiences while cultivating an internal sense of calmness. The ultimate goal, of course, is to generalize these skills from the practice on the mat to everyday life situations. In sum, YBP offer a combination of tools for decreasing allostatic load via vagal afference, with a consequent increase of parasympathetic activation and promotion of self-regulatory mechanisms.

### Integration of Bottom-up and Top-down Processes

From the previous sections it is evident that YBP involve a rich and complex set of both bottom-up physiological and top-down cognitive processes, and there are various ways in which these processes interact and influence each other. Proponents of theoretical frameworks such as allostatic regulation (McEwen and Wingfield, 2003) and the polyvagal perspective (Porges, 2007), emphasize the strong link between visceral regulation and the functioning of the central nervous system, and advocate that there is no real functional separation between the viscera and the brain. Many of the studies of bottom-up/top-down interactions involve risk evaluation and emotional processing, and they outline both bottom-up influences on higher brain functions, and top-down regulation of physiological processes. Crucial structures for the bidirectional signaling between the body and the brain involve the hippocampus and the amygdala, as they process experiences by interfacing with both brainstem areas involved in metabolic regulation, and prefrontal areas involved in attentional control (McEwen and Gianaros, 2011).

The concept of neuroception (Porges, 2003) refers to the contribution of bottom-up processes such as vagal afference, sensory input, and endocrine mechanisms to the detection and evaluation of environmental risk prior to the conscious elaboration by higher brain centers. Vagal afferent information is mediated via the thalamus to the insula, anterior cingulate and prefrontal cortex, which are all involved in emotion regulation (Thayer and Sternberg, 2006). Similarly, it has been shown that afferent input from the heart influences the activity of brain regions involved in emotional, perceptual and attentional processing, and that individual differences in HRV can predict attentional inhibition (Park et al., 2012). Moreover, the secretion of stress hormones can influence the evaluation and memory of threat related events via hormone receptors in the hippocampal formation (McEwen, 2013). Conversely, by detecting and evaluating risk, higher order brain centers modulate autonomic states and the expression of adaptive defensive behaviors. For example, structures such as the amygdala and prefrontal cortex, which are involved in fear-detection, attentional mechanisms, executive function and self-regulatory behaviors (McEwen and Gianaros, 2011), are linked via the vagus nerve to the regulation of metabolic systems (Thayer and Sternberg, 2006). Similarly, temporal regions involved in the perception of biological movement, faces and vocalizations (Adolphs, 2002), can trigger or inhibit physiological responses and affect allostatic load (Porges, 2007). As a result of the bidirectional neurocircuitry involved in stress regulation, the brain can also undergo structural changes over time. In fact, it has been shown that even in otherwise healthy individuals, chronic exposure to stress can alter GMV in the hippocampus, amygdala and prefrontal cortex (Ganzel et al., 2008).

Given that YBP employ both bottom-up and top-down mechanisms, they lend themselves as a method for dynamically exploring the interplay between the body’s stress responses and regulatory systems (Streeter et al., 2012). Physiological stress responses may be elicited by the physical, emotional or mental challenges that arise during the practice. As these occur, both bottom-up breath-related and top-down attention-related processes are constantly employed to counteract them and reinstall a balance within the system. The movement, in turn, allows for these processes to be applied in a dynamic and ecological way, that consequently makes the effects of YBP likely more generalized to everyday life situations.

## Conclusion

In this paper we propose a definition of YBP with the aim of providing a comprehensive theoretical framework applicable within Western science, from which testable scientific hypotheses can be formulated.

We begin by presenting a brief overview of the extant literature investigating the effects of YBP in healthy populations, with a specific focus on physiological parameters, body awareness, self-reported emotional states and stress, and cognitive functioning. We then discuss some of the methodological shortcomings of previous studies, with particular emphasis on the inappropriate selection of experimental populations and control groups, the use of self-report outcome measures, poorly described interventions, mostly neglected investigations of individual component parts of the programs, and the lack of hypotheses about specific neurophysiological and neurocognitive mechanisms underlying the reported effects of YBP. Subsequently, we outline the main component parts of YBP, which commonly consist of a combination of postures or movement sequences, conscious regulation of the breath, and various techniques to improve attentional focus. We believe that a detailed deconstruction of these component parts is essential for their operationalization and for a better understanding of their respective effects. Lastly, we discuss some of the main neurophysiological and neurocognitive processes hypothesized to underlie the mechanisms of change promoted by YBP. We propose that compared to mindfulness-based practices, the rich set of movement, breath and attention components employed in YBP may more directly engage the vagal afferent system as well as BG and cerebellar circuits, with consequent possibly enhanced effects on autonomic, emotional and cognitive regulation.

In sum, we believe in the importance and potential of future research investigating the mechanisms underlying YBP, so that they may be more effectively adapted and applied in various clinical, educational and recreational settings. The theoretical framework presented in our paper is by no means exhaustive, but only intending to represent a starting point from which specific hypotheses for future research can be formulated. We hope that it will inspire further work in the field with the ultimate aim of unveiling the full potential of YBP in modern contexts.

## Conflict of Interest Statement

The authors declare that the research was conducted in the absence of any commercial or financial relationships that could be construed as a potential conflict of interest.
